# Web-Based Graphic Representation of the Life Course of Mental Health: Cross-Sectional Study Across the Spectrum of Mood, Anxiety, Eating, and Substance Use Disorders

**DOI:** 10.2196/16919

**Published:** 2020-01-28

**Authors:** Robin Leora Aupperle, Martin P Paulus, Rayus Kuplicki, James Touthang, Teresa Victor, Hung-Wen Yeh, Tulsa 1000 Investigators, Sahib S Khalsa

**Affiliations:** 1 Laureate Institute for Brain Research Tulsa, OK United States; 2 Oxley College of Health Sciences The University of Tulsa Tulsa, OK United States; 3 Health Services & Outcomes Research Children’s Mercy Hospital Kansas City, MO United States

**Keywords:** mental health, life history, psychosocial factors, depression, anxiety, substance use disorders, eating disorders

## Abstract

**Background:**

Although patient history is essential for informing mental health assessment, diagnosis, and prognosis, there is a dearth of standardized instruments measuring time-dependent factors relevant to psychiatric disorders. Previous research has demonstrated the potential utility of graphical representations, termed *life charts*, for depicting the complexity of the course of mental illness. However, the implementation of these assessments is limited by the exclusive focus on specific mental illnesses (ie, bipolar disorder) and the lack of intuitive graphical interfaces for data collection and visualization.

**Objective:**

This study aimed to develop and test the utility of the Tulsa Life Chart (TLC) as a Web-based, structured approach for obtaining and graphically representing historical information on psychosocial and mental health events relevant across a spectrum of psychiatric disorders.

**Methods:**

The TLC interview was completed at baseline by 499 participants of the Tulsa 1000, a longitudinal study of individuals with depressive, anxiety, substance use, or eating disorders and healthy comparisons (HCs). All data were entered electronically, and a 1-page electronic and interactive graphical representation was developed using the Google Visualization Application Programming Interface. For 8 distinct life epochs (periods of approximately 5-10 years), the TLC assessed the following factors: school attendance, hobbies, jobs, social support, substance use, mental health treatment, family structure changes, negative and positive events, and epoch and event-related mood ratings. We used generalized linear mixed models (GLMMs) to evaluate trajectories of each domain over time and by sex, age, and diagnosis, using case examples and Web-based interactive graphs to visualize data.

**Results:**

GLMM analyses revealed main or interaction effects of epoch and diagnosis for all domains. Epoch by diagnosis interactions were identified for mood ratings and the number of negative-versus-positive events (all *P* values <.001), with all psychiatric groups reporting worse mood and greater negative-versus-positive events than HCs. These differences were most robust at different epochs, depending on diagnosis. There were also diagnosis and epoch main effects for substance use, mental health treatment received, social support, and hobbies (*P*<.001). User experience ratings (each on a 1-5 scale) revealed that participants found the TLC pleasant to complete (mean 3.07, SD 1.26) and useful for understanding their mental health (mean 3.07, SD 1.26), and that they were likely to recommend it to others (mean 3.42, SD 0.85).

**Conclusions:**

The TLC provides a structured, Web-based transdiagnostic assessment of psychosocial history relevant for the diagnosis and treatment of psychiatric disorders. Interactive, 1-page graphical representations of the TLC allow for the efficient communication of historical life information that would be useful for clinicians, patients, and family members.

## Introduction

### Background

Understanding the longitudinal course of mental health is crucial for guiding the clinical conceptualization of mental illness [1]. Psychosocial history is particularly important for illuminating environmental, psychological, and biological factors that may influence an individual’s course of illness. Yet, it is one that psychiatry has struggled to adequately and effectively integrate into explanatory models [2-4]. Current assessments of psychosocial history often rely on unstructured methods, such as open-ended interviews. With the advent of electronic medical records, some clinicians and/or health systems have adopted more structured approaches; however, there remains variability in the quality and type of information contained. Such variability stands in contrast to standardized self-report and diagnostic assessments [5,6], potentially neglecting a nuanced understanding of individual patients in the clinic as well as grouped samples in research studies.

One approach for capturing the complexity of an individual’s illness course is to depict it graphically. Post et al [7] developed the National Institute of Mental Health (NIMH) Life Chart method, involving pictorial demonstrations of manic and depressive episodes of individuals with bipolar disorder [8]. In this approach, data gathered from patient and collateral interviews were incorporated into a visual chart organized according to a chronological timeline, with illustrations of pertinent events (eg, suicide attempts, hospitalizations, and medication changes) and illness severity on a perpendicular axis. The resulting chart consisted of a single image containing the majority of outcomes perceived to be pertinent to their illness course. This material, intended to be shared with the patient, family members, or clinicians, was thought to (1) succinctly identify and communicate the emergence of certain patterns in their illness trajectory, (2) assist therapeutic management decisions via an explicit observational measure (eg, similar to monitoring of blood glucose levels in an individual with diabetes), (3) enhance the psychotherapeutic process by increasing insight into these patterns, and (4) improve continuity of care when transitioning from 1 clinician or health system to another. Life history interviews used in other mental health populations (without graphical representations of data collected) have also been found to increase the detection and diagnosis of mental health disorders [9,10].

Despite the apparent advantages of structured and graphical life history methods, this approach has not been widely adopted within the mental health field mainly because of the amount of time required to collect such information and the technological resources needed to develop software with an intuitive graphical interface. In addition, most existing measures focus exclusively on bipolar disorder, with little regard to the high levels of comorbidity across diagnostic categories [11], healthy/resilient populations [12], or relevant psychosocial contexts (eg, social supports, traumatic events, employments, educational history, or stability of home environment) [13]. Furthermore, current assessments do not readily provide an intuitive graphic representation for clinicians, patients, and family members to gain insight into the individual’s unique psychosocial history.

### Objective

Inspired by the NIMH Life Chart approach for bipolar disorder, the primary goals of this study were to (1) develop a structured approach for obtaining historical information on psychosocial and mental health events across a spectrum of disorders, (2) reveal unique patterns of an individual’s life events in a manner not captured by standard clinical practice, and (3) illustrate individual and group historical information through an interactive graphical medium that could be readily examined by clinicians, patients, and family members. To accomplish these goals, we developed the Tulsa Life Chart (TLC), a structured assessment for graphically representing psychosocial historical data at individual and group levels. To test the feasibility and utility of this approach, we applied the TLC to the first 500 subjects of the Tulsa 1000 project, a longitudinal observational study of treatment-seeking individuals with mental health problems across the categories of major depressive disorder (MDD), anxiety disorders (ANX), eating disorders (ED), and substance use disorders (SUD) as well as those without mental health conditions [14]. We provide case examples with TLC graphical representations to illustrate the potential clinical utility of the TLC at the individual level. We hypothesized that the TLC would demonstrate feasibility and utility in detecting and displaying transdiagnostic differences in psychosocial trajectories across the life span. We also examined diagnostic group differences in trajectories of TLC psychosocial events over time to test specific hypotheses and to provide context from which individual case responses on the TLC could be examined. We hypothesized that all mental health conditions would be associated with more negative life events and stressors (eg, changes in residence, schools, and jobs) and fewer recreational activities relative to healthy comparisons (HCs). Given previous research suggesting that the negative effects of psychosocial stress accumulate over time [15], we hypothesized that these diagnostic group differences would be more apparent in later life periods. In addition, we hypothesized that SUD populations would report greater use of substances, starting in adolescence (consistent with previous research [16]), relative to all other diagnostic and HC groups.

## Methods

### Participants

The TLC was completed as part of the Tulsa 1000 project [14], which included participants aged 18 to 55 years, screened on the basis of several dimensional psychopathology scores: the Patient Health Questionnaire-9 (PHQ-9) [17] score greater than or equal to 10, Overall Anxiety Severity and Impairment Scale (OASIS) [18] score greater than or equal to 8, 10-item Drug Abuse Screening Test (DAST) [19] score greater than or equal to 3, and/or Eating Disorder Screen (SCOFF) [20] score greater than or equal to 2. These scales are commonly used screening questionnaires within research and clinical settings and were used within the Tulsa 1000 project to identify participants experiencing clinically significant symptoms of MDD, ANX, SUD, or ED. HC participants without elevations in symptoms or psychiatric diagnoses were also included. Participants were excluded if they (1) tested positive for drugs of abuse; (2) met criteria for psychotic, bipolar, or obsessive-compulsive disorders; (3) reported a history of moderate-to-severe traumatic brain injury, neurological disorders, or severe or unstable medical conditions; (4) had active suicidal intent or plan; or (5) reported a change in medication dose within 6 weeks of study enrollment. Full inclusion/exclusion criteria are described in the study by Victor et al [14]. These criteria were selected to minimize risks to participants and to address factors that may have confounded multilevel assessments conducted as part of the larger Tulsa 1000 study (including functional magnetic resonance imaging) while also optimizing generalizability to community mental health populations (eg, not excluding for many comorbidities or medication use). Participants were recruited from community mental health clinics and the general community through electronic and print advertisements. The Western Institutional Review Board approved the study. All participants provided written informed consent before completion of the study protocol and were compensated for participation. The authors assert that all procedures contributing to this work comply with the ethical standards of the relevant national and institutional committees on human experimentation and with the Helsinki Declaration of 1975, as revised in 2008 (Trial Registration: ClinicalTrials.gov #NCT02450240).

This study focused on baseline data collected from the first 500 participants of the Tulsa 1000, for which longitudinal data collection is ongoing (recruited from January 5, 2015, to February 22, 2017; the study was conducted at the Laureate Institute for Brain Research, LIBR). TLC data were available for 499 individuals (see table in Multimedia Appendix 1 for the number of participants with data for each epoch). Participants were grouped by Diagnostic and Statistical Manual of Mental Disorders (DSM)–IV or DSM-5 diagnosis as determined by the Mini International Neuropsychiatric Inventory (MINI) [21], including MDD only, ANX only (social anxiety, generalized anxiety, panic, or posttraumatic stress disorder), comorbid MDD and ANX (MDD+ANX), SUDs (recreational drugs, excluding alcohol or nicotine; with or without comorbid ANX/MDD), EDs (with or without comorbid MDD, ANX, or SUDs), and HCs with no psychiatric diagnoses. Sample size, demographic information, and screening measure scores are shown in Multimedia Appendix 2.

### Measures

Development of the TLC was informed by the general approach of the NIMH Life Chart [7,8] and by considering transdiagnostic psychosocial factors thought to contribute to mental health. The TLC was conducted as a structured interview by bachelor’s- or master’s-level research personnel and took approximately 2.5 hours to administer. Training in TLC administration was supervised by a board-certified psychiatrist (SK) and a licensed clinical psychologist (RA), with interviewers presenting each case as part of a weekly, supervised assessment review. In addition to the TLC, all participants completed screening measures and the MINI [21].

The TLC interview assessed birth date, location, and birth complications and then queried specific components from each of the following epochs: (1) birth to elementary school or age 5 years, (2) elementary school or ages 5 to 10 years, (3) middle school or ages 11 to 14 years, (4) high school or ages 15 to 18 years, (5) young adult or ages 18 to 25 years, and (6-8) for every 10 years thereafter to the age of the participant (ie, 25-35, 35-45, and 45-55 years). For each epoch, participants provided an average mood rating (1-10, with 1 being the worst they have ever felt and 10 being the best they have ever felt) as well as the frequency/number, start/end dates, and brief descriptions for each of the following: (1) places lived (residences); (2) schools attended (schools); (3) leisure activities (hobbies); (4) employment (jobs); (5) people they felt close to (people); (6) exposure or experiences with substances; (7) mental health treatments; (8) changes in family structure, for example, birth of a child, marriages, divorces, and so forth (change events); (9) negative (bad); (10) positive (good) events; and (11) any *other* events they felt were important. Participants were asked to rate their mood at the time of each negative, positive, change, and other events on the same 1 to 10 scale. For the TLC, we purposely used a mood rating that spanned positive affect (*best they have ever felt*) and negative affect (*worst they have ever felt*) such that the same scale could be used across different types of events (ie, rather than using measures of general distress). All data were entered electronically using Research Electronic Data Capture (REDCap [22]).

A 1-page electronic and interactive graphical representation was developed by exporting data using the redcap.js client module and creating interactive graphs using JavaScript along with the Google Visualization Application Programming Interface [23]. We provide 4 example TLCs, along with brief case summaries of each. For the purposes of confidentiality, information relating to identifiable patient characteristics and personal history has been modified, and each participant provided consent to use the presented information for publication. Interactive graphs for each case, REDCap forms, and code for creating the interactive graphs are available on GitHub [24].

To gauge user experiences of the TLC, a subset of 338 participants were asked to provide quantitative usability ratings, including the pleasantness of the interview, usefulness for better understanding their mental health, and how likely they would be to recommend the TLC to others. Open-ended questions were subsequently asked to gather qualitative feedback about the TLC. These questions included asking participants to elaborate on what was helpful and unhelpful and what they liked and disliked and to provide any other information they thought we should know about their experience. Interviewers made notes concerning the participants’ responses, and thus, the comments are paraphrased rather than being verbatim responses from participants. The authors identified examples of positive and negative comments that were helpful in considering the potential utility of the TLC as well as potential improvements that could be made. Herein, we report the quantitative and qualitative user feedback obtained from participants. For the qualitative feedback, we provide illustrative excerpts highlighting participants’ subjective experiences with the TLC concerning (1) insight into the trajectory of their mental health symptoms, (2) self-awareness of resiliency, (3) user feedback on the structure of the TLC interview, and (4) negative aspects of the TLC. We also provide a complete listing of all qualitative participant feedback obtained from participants.

### Statistical Analysis

Quantitative analyses focused on evaluating (1) general mood ratings (*mood*), (2) the number of bad minus good events (*bad−good*), (3) the number of drug types exposed to (*substance use*), (4) the number of mental health treatments received (*treatment*), (5) the number of people they felt close to (*people*), and (6) the number of hobbies reported (*hobbies*). The number of residences was entered inconsistently by research personnel and was, therefore, not analyzed. We applied generalized linear mixed effects models (GLMMs) on each outcome measure using all available data from each epoch. Fixed effects included epoch, diagnostic group, age, and sex. The choice of distribution (eg, Poisson vs negative binomial for count data), whether to include epoch-by-group interaction effects, and method of capturing temporal dependency (random subject intercept or first-order autoregressive correlations) resulted in several combinations (see Multimedia Appendix 3). The optimal model was determined by the Akaike Information Criterion (AIC). Statistical analyses were conducted in R (version 3.5.0) [25] using the glmmTMB [26] package for GLMMs and the sjPlot [27] package for visualization of parameter estimates and conducting the likelihood ratio tests to test for significance of fixed effects. In addition to providing *P* values for significance testing, incidence rate ratios in log scale, that is, fixed effect coefficients from GLMM, were displayed to provide an indication of the magnitude of the effects identified. The R code used for analysis can be found through the Open Science Framework [28].

## Results

### Individual Tulsa Life Chart Examples

**Figure 1:**
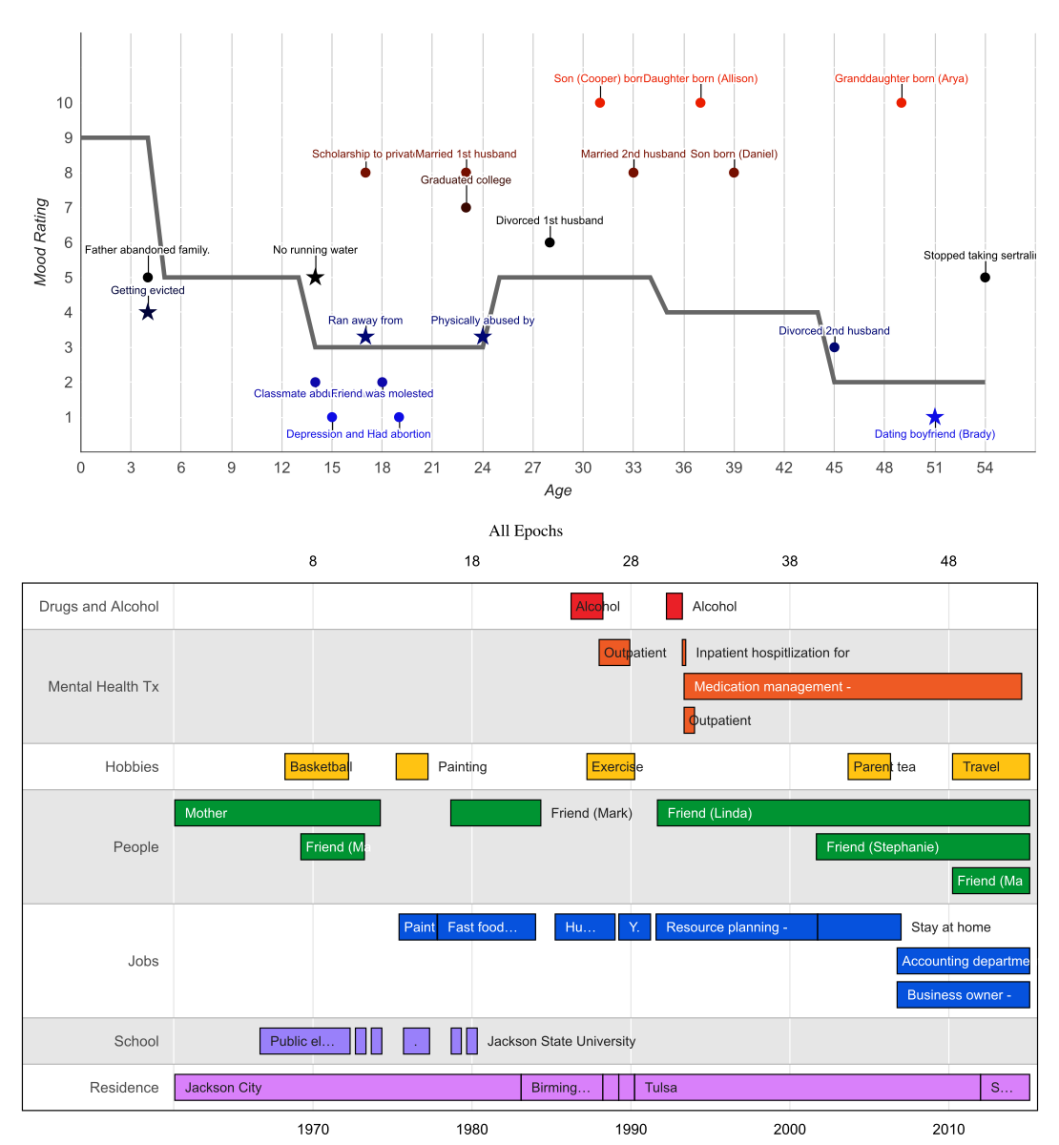
Image from the interactive Tulsa Life Chart for Case 1, from the major depressive disorder comorbid with anxiety disorder diagnostic group.

Case 1 is included to illustrate the TLC at the individual level (Figure 1). Cases 2 to 4 are presented in Multimedia Appendix 4. Interactive TLCs for all 4 cases are available at [24]. In each graph, a top panel displays the average mood rating for each epoch (gray line). Positive, negative, change, and other events are plotted such that the vertical location represents the magnitude of the event-related mood rating, and the horizontal location represents the event date. In the interactive graphs, scrolling the cursor over each event displays additional details (eg, description, rating, and duration). A bottom panel displays information for each of the life components assessed (substance use, treatment, people, hobbies, jobs, schools, and residences), with the length of each box representing the event duration. A left-hand pane allows the user to selectively display/hide aspects of each graph. At the top of the page, a dropdown menu titled *Overall Graphs* displays individual case information alongside averaged group data, providing additional context for evaluation and interpretation.

Case 1 is a 54-year-old African American female. She was adopted at birth and raised by her adoptive parents in Jackson City, FL. At age 5 years, her adopted father reportedly took all the money out of their bank account and left. Her mother subsequently struggled financially and was evicted from multiple houses. She lived in a mobile home with no running water or electricity throughout high school. At age 14 years, a classmate was abducted and raped in an area near her school. It was about this age when she first began experiencing depression and suicidal thoughts. At age 17 years, she ran away from home and stayed with friends for about 1 month. She managed to obtain an academic scholarship to attend a private high school, and while there, she completed the requirements for her last 2 years in 1 year. She reported having an abortion at age 19 years. She indicated that she barely managed to obtain passing grades throughout college and graduated at age 23 years. Soon after, she married her first husband and began working as a human resources administrator. Her husband was physically abusive throughout their marriage. At age 25 years, she began drinking alcohol daily (1-2 glasses of wine). At age 28 years, she got divorced, quit her administrative job, and started working as a yoga instructor. Soon thereafter, she sought outpatient therapy for anxiety. At age 30 years, she moved to Tulsa, OK, and started working for a cellular phone company in the resource planning department. After experiencing sexual harassment from a supervisor, she resumed drinking alcohol daily (1-2 glasses of wine per night). At age 32 years, she was hospitalized in a psychiatric facility for 2 weeks after presenting to her local emergency department reporting suicidal ideation. During her inpatient stay, she was diagnosed with MDD and prescribed sertraline and bupropion. After discharge, she attended outpatient psychotherapy weekly for approximately 6 months and received medication management from a psychiatrist. At age 33 years, she married her second husband with whom she had 3 children. After her last son was born, she quit working to stay at home with her children. She divorced her second husband at age 45 years. Since then, she has worked in accounting at a hardware store and runs her own business. At age 51 years, she moved to Skiatook, OK, and met her current boyfriend, with whom she enjoys traveling. Six months ago, she stopped taking her sertraline and bupropion because of side effects (“feeling like a zombie”). Her anxiety and depression subsequently increased and continues to the present time.

As observed within the TLC graphs, case 1’s mood decreased in childhood when her father left her family but continued to worsen into young adulthood and never completely recovered (despite engaging in treatment). She did not report many hobbies in her past but consistently obtained long-term employment at several places (each lasting >2 years). Although she did not report being close to many people in the past, she named 3 friends she currently feels close to. She has had very little exposure to drugs of abuse, with the exception of alcohol. Her scores on screening measures were as follows: SCOFF=1, PHQ-9=14, OASIS=11, and DAST=0, suggesting elevations in anxiety (severe) and depression (moderate) symptoms. Through the MINI, she was diagnosed with MDD, recurrent; generalized anxiety disorder; and panic disorder without agoraphobia.

### Usability Ratings

Participants reported their experience completing the TLC as pleasant, on average (see Table 1). They also indicated that the TLC was somewhat helpful for better understanding their mental health and that they would be somewhat to moderately likely to recommend the TLC to others. Textbox 1 provides patient perspectives of the TLC, as illustrated through individual feedback comments provided by participants. All deidentified, qualitative participant feedback is provided in Multimedia Appendix 5.

**Table 1:** Average responses to the feedback questionnaire concerning the Tulsa Life Chart.

Question	Rating (scale 1-5)	
	Mean (SD)	Median
		
How was your experience of completing the life chart? (*Anchors: Very unpleasant, unpleasant, neutral, pleasant, very pleasant)*	3.73 (1.10)	4
How helpful was completing the life chart in better understanding your mental health? (*Anchors: Not at all, slightly, somewhat, moderately, extremely)*	3.07 (1.26)	3
How likely would you be to recommend the life chart to others with mental health concerns? (*Anchors: Not at all, slightly, somewhat, moderately, extremely)*	3.42 (0.85)	3

**Perspectives about the Tulsa Life Chart by individual participants.**

As part of the Tulsa Life Chart (TLC) feedback questionnaire, 338 participants were asked to provide qualitative input concerning their experience with the interview. Below are paraphrased excerpts from these responses, organized by themes related to (1) the insight they experienced, (2) observations of resilience, (3) comments about the structure of the TLC, and (4) comments concerning potential negative aspects of the TLC.

**Insight**

- “The lifecharting allowed me to look at each event that has happened in my life and think about how it affected me. It gave me a base to work from in my therapy.”
- “It took one question to hash open my upbringing, and it made me think that if one question could open my mind that much then seeing a psychiatrist could be helpful. It makes it easier to heal when you can understand things, when you know the why behind something. Now I feel like I can move on easier and put things to rest.”
- “It helped me to recall certain life events and see a correlation between those events and my substance use, or thought patterns emerging that led to my substance use.”
- “[The lifechart] helped me understand my depression a lot more because I’ve never sat down and reviewed the different events in my life that may have contributed to it.”
- “I would like to keep up with this, maybe informally through a journal or documenting in some way.”

**Resilience**

- “Reflecting on all the memories that make up my life changed my perspective on why I’ve struggled with depression but also made me appreciate how resilient I have been in spite of those things.”
- “Lifecharting was helpful to realize how much I have conquered in my life and helped me understand how resilient I have been.”
- “I thought it was helpful to see how strong I was and how I overcame the bad things that happened.”

**Structure**

- “I enjoyed talking about both the ‘pros and cons’ of each life period and talking about both the good and bad.”
- “I liked the structure of the interview; having life broken down into each section made it easy to remember.”
- “I liked life charting because it lets you see your life through a window, it allowed me to do great reflection.”
- “Going through my life in a linear way puts things in a more organized perspective.”

**Negative aspects**

- “I did not like how long the interview was; the process was overwhelming at times.”
- “I wish I would have known ahead of time what the questions were.”
- “I felt frustrated when I wasn’t able to give precise dates.”
- “I did not like having to talk about the negative events that occurred throughout my life.”
- “I did not like having to share my story with a stranger.”

### Group-Level Analysis

**Figure 2:**
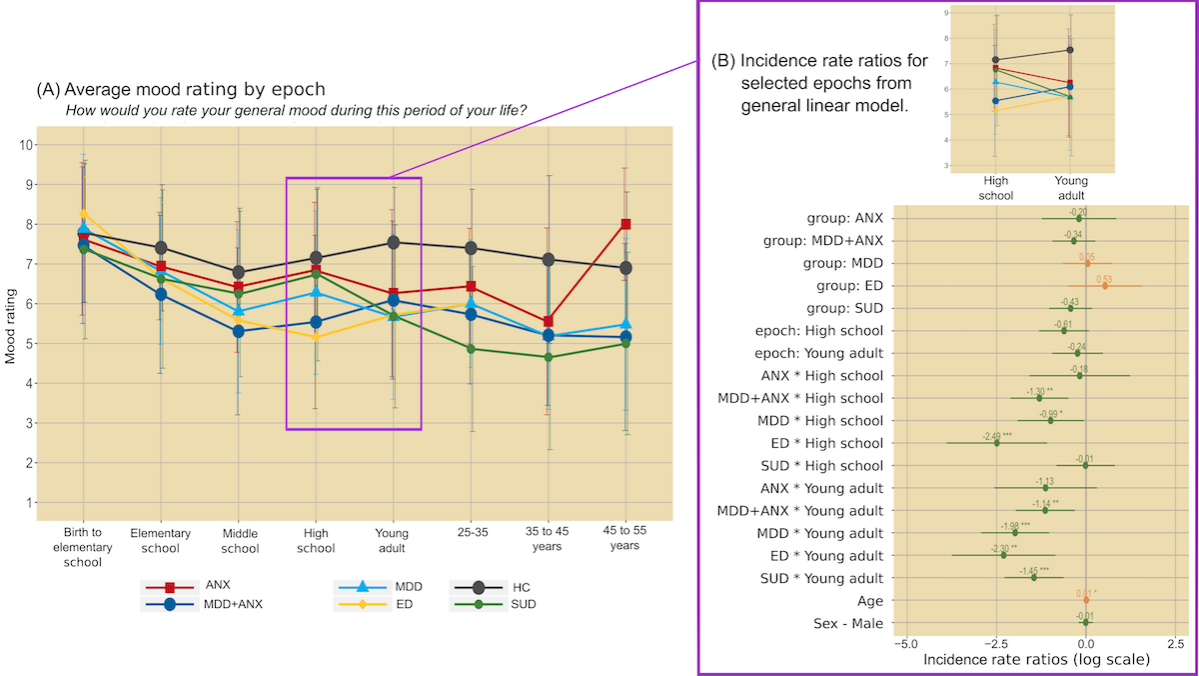
Average mood rating by epoch (left panel) and regression coefficients from generalized linear mixed effects models (right panel). Error bars on the left graph represent standard deviation; error bars on the right graph represent 95% CIs; positive values in orange, negative values in green), with *P* value thresholds noted with *.05, **.01, and ***.001. ANX: anxiety disorder; ED: eating disorder; HC: healthy comparison; MDD: major depressive disorder; MDD+ANX: major depressive disorder comorbid with anxiety disorder; SUD: substance use disorder.

**Figure 3:**
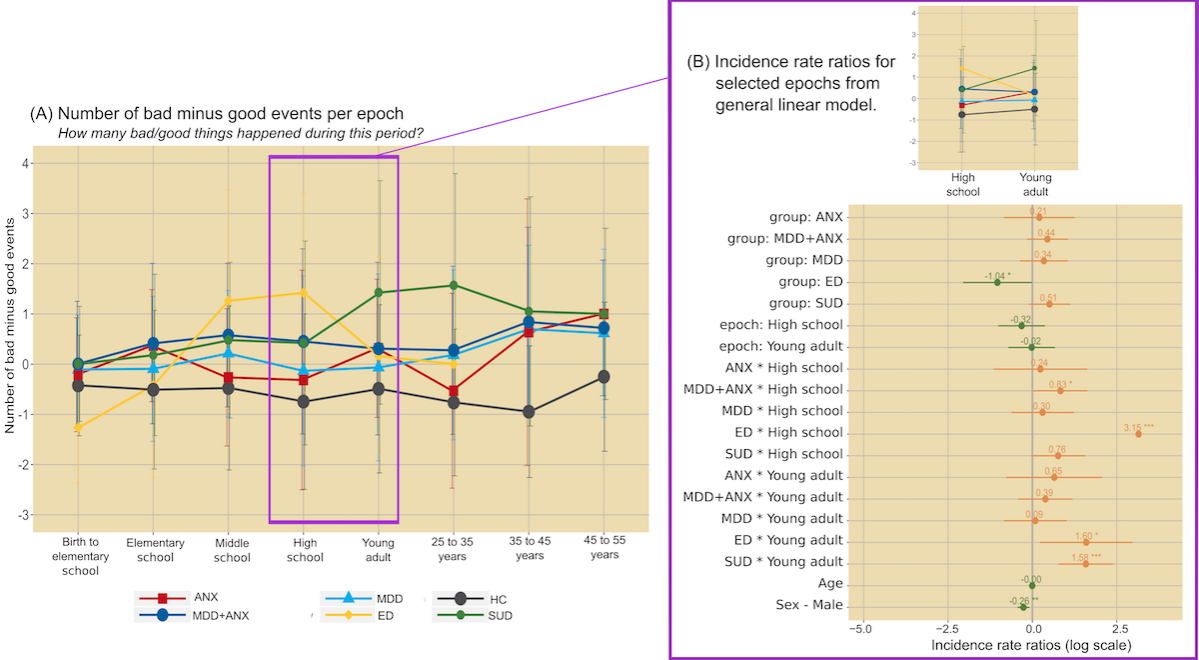
Average number of bad minus good events reported by epoch (left panel) and regression coefficients from generalized linear mixed effects models (right panel). Error bars on the left graph represent standard deviation; error bars on the right graph represent 95% CIs (positive values in orange; negative values in green) and *P* value thresholds noted with *.05, **.01, and ***.001. ANX: anxiety disorder; ED: eating disorder; HC: healthy comparison; MDD: major depressive disorder; MDD+ANX: major depressive disorder comorbid with anxiety disorder; SUD: substance use disorder.

**Figure 4:**
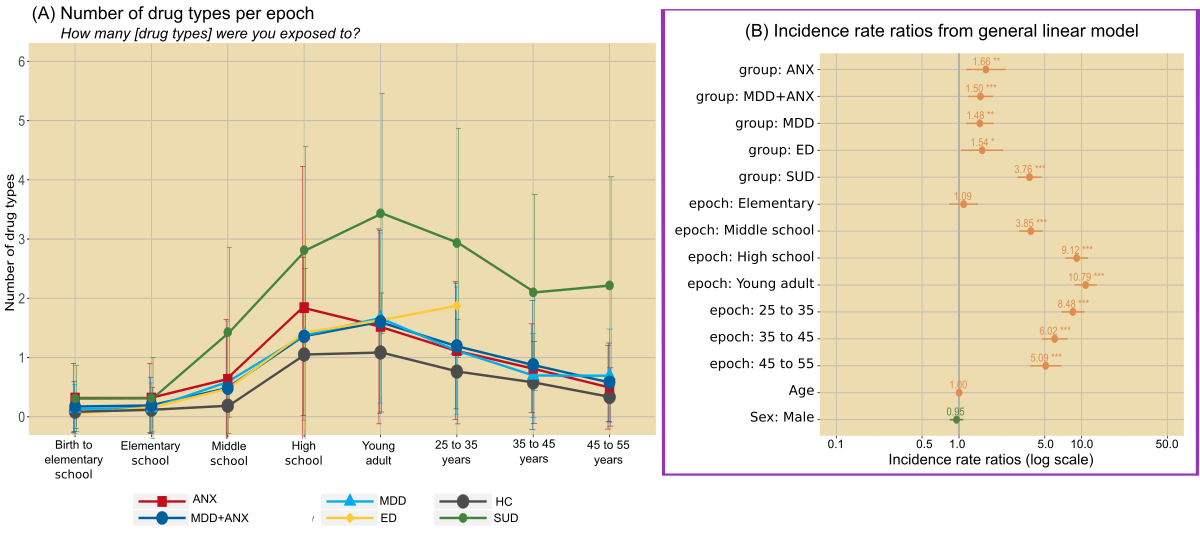
Average number of drug types exposed to by epoch (left panel) and regression coefficients from generalized linear mixed effects models (right panel). Error bars on the left graph represent standard deviation; error bars on the right graph represent 95% CIs (positive values in orange; negative values in green) and *P* value thresholds noted with *.05, **.01, and ***.001. ANX: anxiety disorder; ED: eating disorder; HC: healthy comparison; MDD: major depressive disorder; MDD+ANX: major depressive disorder comorbid with anxiety disorder; SUD: substance use disorder.

For GLMM analysis of mood ratings, the model selected by AIC identified epoch by diagnosis interactions (χ^2^_35_=118.0; *P*<.001). Specifically, MDD and MDD+ANX reported reduced mood ratings starting in elementary school, which became more pronounced in later epochs (Figure 2). ED also reported reduced mood ratings, but with the most robust decreases observed in high school years. For SUD, decreases in mood ratings did not occur until young adulthood. For bad-good events, the model selected by AIC also identified epoch by diagnostic group interactions (χ^2^_35_=339.0; *P*<.001; Figure 3). Specifically, ANX, MDD, and MDD+ANX exhibited greater bad-good event ratios compared with HCs, most robustly in later epochs (ie, the 35-45 years age range). The ED group exhibited the most bad-good events in middle and high school years. For SUD, the most bad-good events occurred during young adulthood and ages 25 to 35 years. For substance use exposure, the model selected by AIC indicated diagnosis (χ^2^_5_=210.0; *P*<.001) and epoch main effects (χ^2^_7_=1169.4; *P*<.001), characterized by the greatest level of substance use reported for all psychiatric groups (but most robustly for SUD) compared with HCs and for high school and young adult epochs (see Figure 4).

As shown in Multimedia Appendix 3, the models selected by the AIC for mental health treatment, social support, and hobbies indicated diagnosis and epoch main effects. Greater treatment seeking was observed for all psychiatric groups compared with HCs and for later epochs (Multimedia Appendix 3 and Figure 1). Less social support was observed for MDD, MDD+ANX, and SUD (but not ED) and for elementary through young adulthood (Multimedia Appendix 3 and Figure 2). Less number of hobbies were observed for MDD and SUD, and the greatest number of hobbies were reported for elementary to young adulthood (Multimedia Appendix 3 and Figure 3).

## Discussion

### Principal Findings

We developed the TLC as an electronic, structured method for assessing and longitudinally representing historical life events across the spectrum of depressive disorder, ANX, ED, and SUD, at individual and group levels, with the long-term goal of improving conceptualizations concerning the development, maintenance, and treatment of mental health by clinicians, patients, and family members. Within a large, transdiagnostic mental health sample, we used the TLC to identify how historical trajectories of psychosocial factors are influenced by life epoch and diagnosis, demonstrating the feasibility of the TLC through case examples, interactive TLC graphs, and patient feedback.

As illustrated in the cases provided, the TLC comprehensively communicates psychosocial information through a 1-page, Web-based graphical interface. For clinicians in practice settings affording limited face time with patients [29], the TLC could provide a quick, easily reviewable *fingerprint* of a patient’s life, displaying in a single page the highs, lows, and major transitions. It also allows the viewer to modify what aspects of the TLC to view, depending on their needs, and to view the mood trajectory of 1 patient concurrently with the average of psychiatric comparison groups. The TLC could be useful for considering symptom onset and trajectory, thereby informing diagnosis and treatment selection, or facilitating deeper follow-up questions concerning the different domains assessed (eg, stability of early life, trauma history, and substance use). For psychologists or other licensed psychotherapy providers, the TLC could be integral for informing psychosocial interventions. For example, identifying hobbies individuals used to enjoy, positive events previously experienced, and sources of social support could be helpful for activity scheduling (eg, for behavioral activation therapy) [30]. As evidenced by participants’ feedback, the TLC could provide therapeutic value in and of itself by enhancing self-awareness concerning links between life events, behaviors, and consequences, which could potentially improve motivation to engage with treatment. As self-monitoring is a cornerstone of most cognitive behavioral interventions, the TLC could provide a common platform from which to monitor mood fluctuations and associated events or behaviors. Given the Web-based platform, the TLC also represents a modifiable assessment instrument that could easily incorporate additional information to meet the individual needs of a clinician and patient (eg, to record cognitions, physical sensations, or other specific symptomatology).

By administering the TLC to a large, transdiagnostic mental health sample, we provided information concerning normative trajectories and potential differences in these trajectories by psychiatric diagnoses. In general, people in this study reported worse mood and more negative (vs positive) life events for later epochs, conflicting somewhat with previous work suggesting negative affect to peak around 20 to 40 years of age [31]. The number of hobbies and social support peaked from elementary through young adulthood and then exhibited a continuous decline. As expected, greater substance use was reported during young adulthood [16,32]. Numerous differences were also observed among the different psychiatric groups. These results support the plethora of research pointing to negative life events as precursors for mental health disorders [33,34] and the increased likelihood of substance use across mental health disorders [35,36]. Patients with MDD, MDD+ANX, and ANX reported increases in negative life events and decreased mood starting as early as elementary school, suggesting mood disturbances may often preexist the young adulthood age in which these disorders are usually diagnosed [16]. For SUD, decreased mood ratings were not observed until young adulthood, consistent with the most common age of onset [16]. Although all psychiatric groups (other than EDs) reported less social support, only MDD and SUD reported fewer hobbies than HCs. The decreases in leisure activities for MDD and SUD likely relate to changes in reward sensitivity and motivation that are hallmarks of these disorders [37,38]. Individuals with ED and ANX (with or without MDD) may retain motivation for such activities or even be motivated to engage in activities as a way of avoiding feared negative outcomes (eg, failure) [39].

### Limitations and Future Directions

Given the potential positive benefits of the TLC endorsed by participants during the usability assessment, it is worth considering the pathway for feasible implementation and potential limitations and obstacles. The current version of the TLC was administered by bachelor’s- or master’s-level assessors and took 2 to 3 hours to complete. Although this is more cost-efficient than assessments requiring administration by licensed clinicians, it is still rather resource intensive. The duration of the interview, difficulty sharing personal details with the interviewer, and difficulty recalling events at the time of the interview were highlighted as potential negative aspects of the TLC by some participants. A self-administered version could optimize efficiency, help to facilitate broad adoption by clinicians, and potentially address some of these participant concerns. A recent empirical study of NIMH Life Chart data in individuals with bipolar disorder reported similar results regardless of clinician administration or self-administration, supporting the potential for such an approach [40]. To optimize access and continuity of care, the TLC could be hosted on a website for patients to complete on their own, with different clinicians given access as needed. However, this raises potential security and privacy concerns that would need to be addressed with information technology and informed consent methods. It should also be recognized that recall of past events and mood ratings could be influenced by recency effects, current mood, or other unknown factors [41,42]. Thus, the TLC may reflect a patient’s perception of their life history, rather than a factual historical account. However, it is possible that after a patient has established a retrospective life chart, they could continually update the TLC prospectively as a momentary assessment tool [43], thus enhancing the reliability of information gathered and allowing for mutual evaluation of trajectories by patient and clinician over time.

The next steps in the development of the TLC are to improve the user experience of individual patients, to investigate the potential clinical utility of this tool from the health care provider perspective, and to enhance the potential for acceptability, scalability, and integration into health care settings by patients and providers. Over the short term, this will likely include (1) developing an interactive self-report version of the TLC that reduces completion time, reduces the personnel cost associated with administration, and increases the ability to deploy the TLC to different clinical settings; and (2) obtaining feedback from clinicians concerning the potential clinical utility of the TLC (eg, by asking them to provide the TLC to a subset of patients and assessing whether the information provided in the TLC improved their clinical understanding of the patient or improved their treatment delivery). In this context, clinicians could include providers across the health care spectrum, such as psychiatrists, psychologists, psychotherapists, primary care physicians, and health care extenders, including nurses, social workers, and medical assistants. Next steps over the long term include (1) identifying ways to link or embed the TLC within the electronic health records of health systems (eg, Epic and Cerner), which could enhance the clinical utility of the TLC for providers across the health care spectrum; and (2) exploring ways in which the TLC might be personalized toward life experiences, such as enabling the linking of nonmedical data with social media websites (eg, perhaps allowing for linking of important events to uploaded pictures, further facilitating the tracking of important life events).

### Generalizability of Findings

In regard to the generalizability of these findings, the current sample consisted of individuals recruited from a community-based sample reporting clinically significant mental health symptoms related to ANX, depressive disorder, SUD, or ED, the latter of which was underrepresented. The sample was somewhat overrepresented in regard to white and Native American populations and underrepresented in regard to Hispanic and Latino, black and African American, and other minority populations, compared with the broader US population [44]. The average family income for the sample was relatively similar to national averages [45], but there were substantial differences in family income between participant groups (with the substance use group reporting the lowest levels of income overall). Future work is needed to establish whether these group-level findings provide meaningful comparison data for individuals with different racial or ethnic backgrounds, income levels, or symptom profiles.

### Conclusions

Understanding a patient’s life history is essential for informing their mental health diagnosis and treatment planning. Clinicians use historical interviews as a way to gather facts and information, to better understand the patient’s trajectory of symptoms and experiences, and to build rapport by communicating that understanding back to patients [46]. The TLC described herein provides 1 strategy for combining the benefits of structured assessments with that of a more clinically oriented and personalized interview. Furthermore, the TLC allows for a 1-page graphical representation of an individual’s life history, providing an efficient method for reviewing a multitude of other psychosocial factors often considered important in the development and treatment of psychiatric disorders. The TLC offers a life-charting method that is relevant across psychiatric conditions and has the potential for informing and enhancing diagnosis and treatment.

## Appendix

Multimedia Appendix 1 Number of participants with Tulsa Life Chart data from each epoch.

Multimedia Appendix 2 Demographic information and symptom severity of study sample.

Multimedia Appendix 3 Supplemental results from generalized linear mixed-effects models examining epoch and diagnostic effects on Tulsa Life Chart categories.

Multimedia Appendix 4 Supplemental examples of individual cases assessed using the Tulsa Life Chart.

Multimedia Appendix 5 Participant feedback concerning their experience completing the Tulsa Life Chart.

## Abbreviations

AIC: Akaike Information Criterion
ANX: anxiety disorder
DAST: Drug Abuse Screening Test
DSM: Diagnostic and Statistical Manual of Mental Disorders
ED: eating disorder
GLMM: generalized linear mixed effects models
HC: healthy comparisons
LIBR: Laureate Institute for Brain Research
MDD: major depressive disorder
MDD+ANX: comorbid major depressive disorder and anxiety disorders
MINI: Mini International Neuropsychiatric Inventory
NIMH: National Institute of Mental Health
OASIS: Overall Anxiety Severity and Impairment Scale
PHQ-9: Patient Health Questionnaire-9
REDCap: Research Electronic Data Capture
SUD: substance use disorder
TLC: Tulsa Life Chart
