# Supplementary material for: Web-Based Graphic Representation of the Life Course of Mental Health: Cross-Sectional Study Across the Spectrum of Mood, Anxiety, Eating, and Substance Use Disorders
Source: JMIR Ment Health. 2020 Jan 28;7(1):e16919. doi: 10.2196/16919 (PMC7013650; doi:10.2196/16919)
Supplement: Multimedia Appendix 1 [file mental_v7i1e16919_app1.docx]

**Multimedia Appendix, Table 1. Number of participants with Tulsa Life Chart data from each epoch.**

| Group | Birth to elementary | Elementary  years | Middle school | High school  years | Young adult  years | Age:  25 to 35 | Age:  35 to 45 | Age:  45 to 55 |
| --- | --- | --- | --- | --- | --- | --- | --- | --- |
| Eating+ (ED) | 19 | 19 | 19 | 19 | 19 | 8 | 2 | 1 |
| Healthy comparisons (HC) | 59 | 59 | 59 | 59 | 59 | 38 | 19 | 12 |
| Anxiety (ANX) | 19 | 19 | 19 | 19 | 19 | 17 | 11 | 2 |
| Depression and anxiety (MDD+ANX) | 168 | 168 | 168 | 168 | 168 | 123 | 81 | 43 |
| Depression (MDD) | 75 | 75 | 75 | 75 | 75 | 60 | 43 | 26 |
| Substance+ (SUD) | 159 | 159 | 159 | 159 | 159 | 135 | 60 | 23 |
| Totals | 499 | 499 | 499 | 499 | 499 | 381 | 216 | 107 |

**This is a Multimedia Appendix to a full manuscript entitled “Web-Based Graphic Representation of the Life Course of Mental Health: A Cross-Sectional Study Across the Spectrum of Mood, Anxiety, Eating, and Substance Use Disorders”**
