# Supplementary material for: Web-Based Graphic Representation of the Life Course of Mental Health: Cross-Sectional Study Across the Spectrum of Mood, Anxiety, Eating, and Substance Use Disorders
Source: JMIR Ment Health. 2020 Jan 28;7(1):e16919. doi: 10.2196/16919 (PMC7013650; doi:10.2196/16919)
Supplement: Multimedia Appendix 2 [file mental_v7i1e16919_app2.docx]

**Multimedia Appendix, Table 2. Demographic information and symptom severity of study sample.**

|  | | HC  (N=59) | | | ANX  (N=19) | | DEP+ANX  (N=168) | | | DEP  (N=75) | | | ED  (N=19) | | | SUD  (N=159) | | | p-value | |
| --- | --- | --- | --- | --- | --- | --- | --- | --- | --- | --- | --- | --- | --- | --- | --- | --- | --- | --- | --- | --- |
| Age M (SD) | 31.3 | | (11.1) | 36.2 | | (9.1) | | 34.2 | (11.4) | | 36.8 | (11.6) | | 26.0 | (8.9) | | 34.1 | (9.9) | | 0.002 |
| Male, N (%) | 29 | | (49%) | 5 | | (26%) | | 43 | (26%) | | 24 | (32%) | | 2 | (11%) | | 74 | (77%) | | <0.001 |
| Ethnicity, N (%) |  | |  |  | |  | |  |  | |  |  | |  |  | |  |  | | 0.832 |
| Hispanic or Latino | 4 | | (7%) | 0 | | (0%) | | 7 | (4.2%) | | 0 | (0%) | | 0 | (0%) | | 1 | (1%) | |  |
| Not Hispanic or Latino | 54 | | (92%) | 19 | | (100%) | | 160 | (95%) | | 70 | (93%) | | 19 | (100%) | | 148 | (93%) | |  |
| Unspecified | 1 | | (2%) | 0 | | (0%) | | 1 | (1%) | | 3 | (4%) | | 0 | (0%) | | 4 | (3%) | |  |
| Race, N (%) |  | |  |  | |  | |  |  | |  |  | |  |  | |  |  | | 0.004 |
| White | 46 | | (78%) | 17 | | (90%) | | 119 | (71%) | | 56 | (76%) | | 18 | (95%) | | 92 | (58%) | |  |
| American Indian or Alaska Native | 1 | | (2%) | 1 | | (5%) | | 6 | (4%) | | 3 | (4%) | | 0 | (0%) | | 21 | (13%) | |  |
| Black or African-American | 2 | | (3%) | 0 | | (0%) | | 12 | (7%) | | 8 | (11%) | | 0 | (0%) | | 10 | (6%) | |  |
| Asian or Pacific Islander | 2 | | (3%) | 0 | | (0%) | | 1 | (1%) | | 1 | (1%) | | 0 | (0%) | | 1 | (1%) | |  |
| Middle-Eastern | 0 | | (0%) | 0 | | (0%) | | 0 | (0%) | | 0 | (0%) | | 0 | (0%) | | 0 | (0%) | |  |
| More than one race | 6 | | (10%) | 1 | | (5%) | | 29 | (17%) | | 5 | (7%) | | 1 | (5%) | | 32 | (20%) | |  |
| "Other", unspecified | 2 | | (3%) | 0 | | (0%) | | 1 | (1%) | | 1 | (1%) | | 0 | (0%) | | 2 | (1.3%) | |  |
| Education, N (%) |  | |  |  | |  | |  |  | |  |  | |  |  | |  |  | |  |
| Less than high school | 0 | | (0%) | 0 | | (0%) | | 10 | (6%) | | 2 | (3%) | | 1 | (5%) | | 35 | (22%) | |  |
| High school degree or equivalent (GED) | 8 | | (14%) | 4 | | (21%) | | 28 | (17%) | | 6 | (8%) | | 3 | (16%) | | 52 | (33%) | |  |
| Some college, no degree | 24 | | (41%) | 5 | | (26%) | | 60 | (36%) | | 31 | (42%) | | 6 | (32%) | | 45 | (28%) | |  |
| Associate’s degree | 4 | | (7%) | 3 | | (16%) | | 22 | (13%) | | 11 | (15%) | | 1 | (5%) | | 18 | (11%) | |  |
| Bachelor’s degree | 17 | | (29%) | 6 | | (32%) | | 36 | (22%) | | 19 | (26%) | | 5 | (26%) | | 8 | (5%) | |  |
| Graduate degree (i.e., masters or doctorate) | 6 | | (10%) | 1 | | (5%) | | 10 | (6%) | | 4 | (5%) | | 3 | (16%) | | 1 | (1%) | |  |
| Income, M (SD) | $56,597 | | ($46,842) | $54,070 | | (56973) | | $55,476 | (90,617) | | 48,282 | (40,203) | | 97,833 | (89,591) | | $26,254 | (55,859) | | <0.001 |
| PHQ-9, M (SD) | 0.90 | | (1.36) | 7.53 | | (5.07) | | 12.86 | (5.13) | | 13.15 | (4.48) | | 12.68 | (7.06) | | 6.49 | (5.68) | | <0.001 |
| OASIS | 1.31 | | (1.88) | 10.47 | | (2.41) | | 10.60 | (3.13) | | 7.76 | (3.46) | | 9.68 | (4.30) | | 5.78 | (4.66) | | <0.001 |
| DAST | 0.12 | | (0.38) | 0.63 | | (1.16) | | 0.61 | (1.22) | | 0.81 | (1.81) | | 1.06 | (2.26) | | 7.48 | (2.20) | | <0.001 |
| SCOFF | 0.08 | | (0.28) | 0.58 | | (0.90) | | 1.19 | (1.27) | | 0.97 | (1.13) | | 3.32 | (1.53) | | 0.57 | (0.90) | | <0.001 |

Abbreviations: HC, Healthy Comparison; ANX, anxiety disorder without major depressive disorder; DEP, major depressive disorder without anxiety diagnoses; DEP+ANX, major depressive disorder with an anxiety diagnoses; ED, eating disorder with or without comorbid diagnoses; SUD, substance use disorder with or without comorbid diagnoses; PHQ-9, Patient Health Questionnaire; OASIS, Overall Anxiety Severity and Impairment Scale; DAST, Drug Abuse Screening Test; SCOFF, eating disorders screening questionnaire.

**This is a Multimedia Appendix to a full manuscript entitled “Web-based graphic representation of the life course of mental health: A cross-sectional study across the spectrum of mood, anxiety, eating, and substance use disorders.**”
