# Supplementary material for: Web-Based Graphic Representation of the Life Course of Mental Health: Cross-Sectional Study Across the Spectrum of Mood, Anxiety, Eating, and Substance Use Disorders
Source: JMIR Ment Health. 2020 Jan 28;7(1):e16919. doi: 10.2196/16919 (PMC7013650; doi:10.2196/16919)
Supplement: Multimedia Appendix 3 [file mental_v7i1e16919_app3.docx]

**This is a Multimedia Appendix to a full manuscript entitled “Web-based graphic representation of the life course of mental health: A cross-sectional study across the spectrum of mood, anxiety, eating, and substance use disorders.**

Additional information concerning the Tulsa Life Chart (TLC) assessment instrument and analyses used for the current study is available through open source websites. Specifically, interactive Graphs for each case, REDCap forms, and code for creating the interactive TLC graphs, are available on GitHub at <https://laureate-institute-for-brain-research.github.io/tlc/>. R code used for analysis can be found at <https://osf.io/58h9r/>.

**Supplemental figures for group-level analyses:**

**
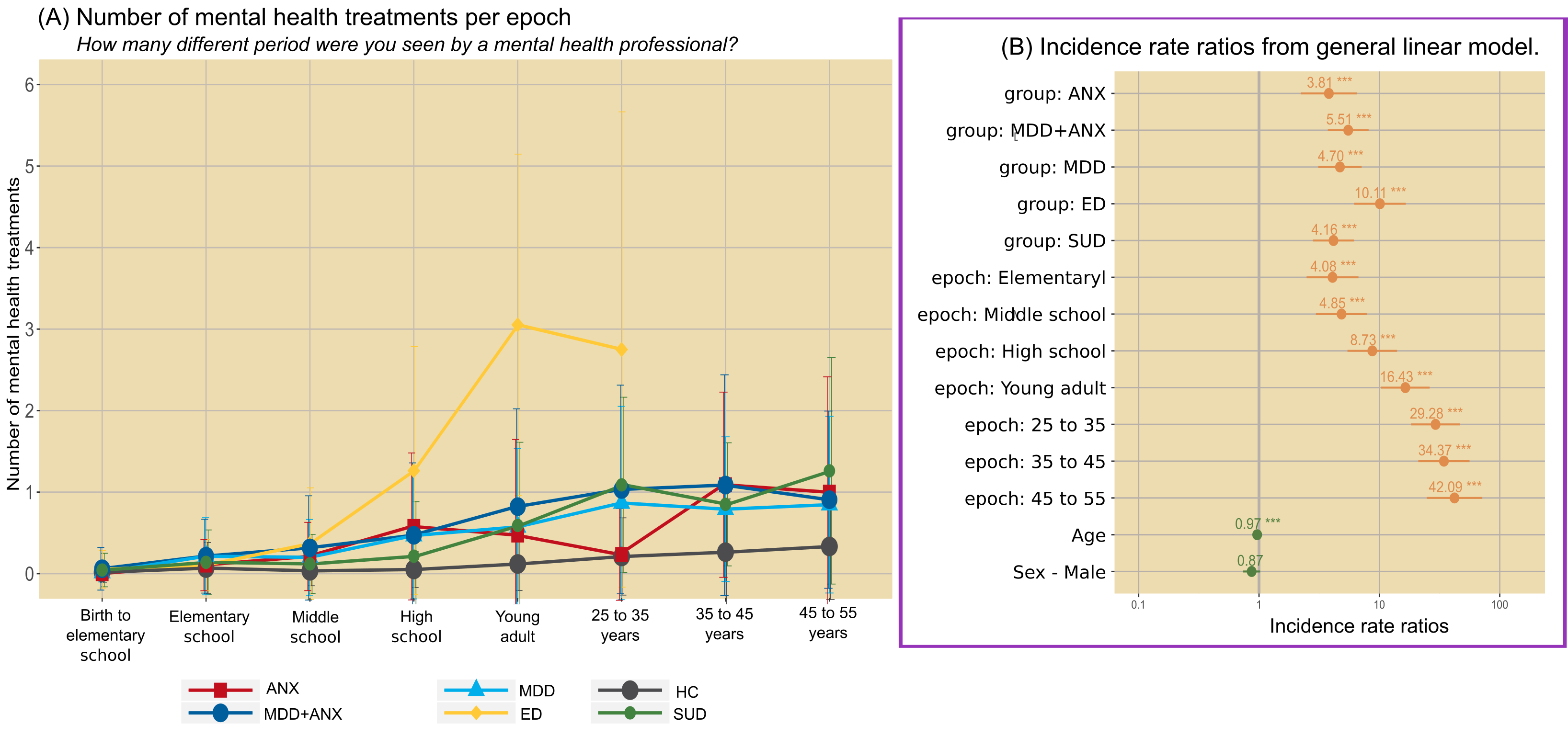
**

**Multimedia Appendix 3, Figure 1. Average number of mental health treatments received by epoch and regression coefficients from generalized linear mixed-effects models.** On the left are the average number of mental health treatments received for each diagnostic group by epoch, with error bars representing standard deviation. On the right side are the regression coefficients obtained from the from generalized linear mixed-effects models identified by Akaike Information Criterion (AIC), with error bars representing 95% confidence intervals and p-value thresholds noted with *.05, **.01, ***.001.


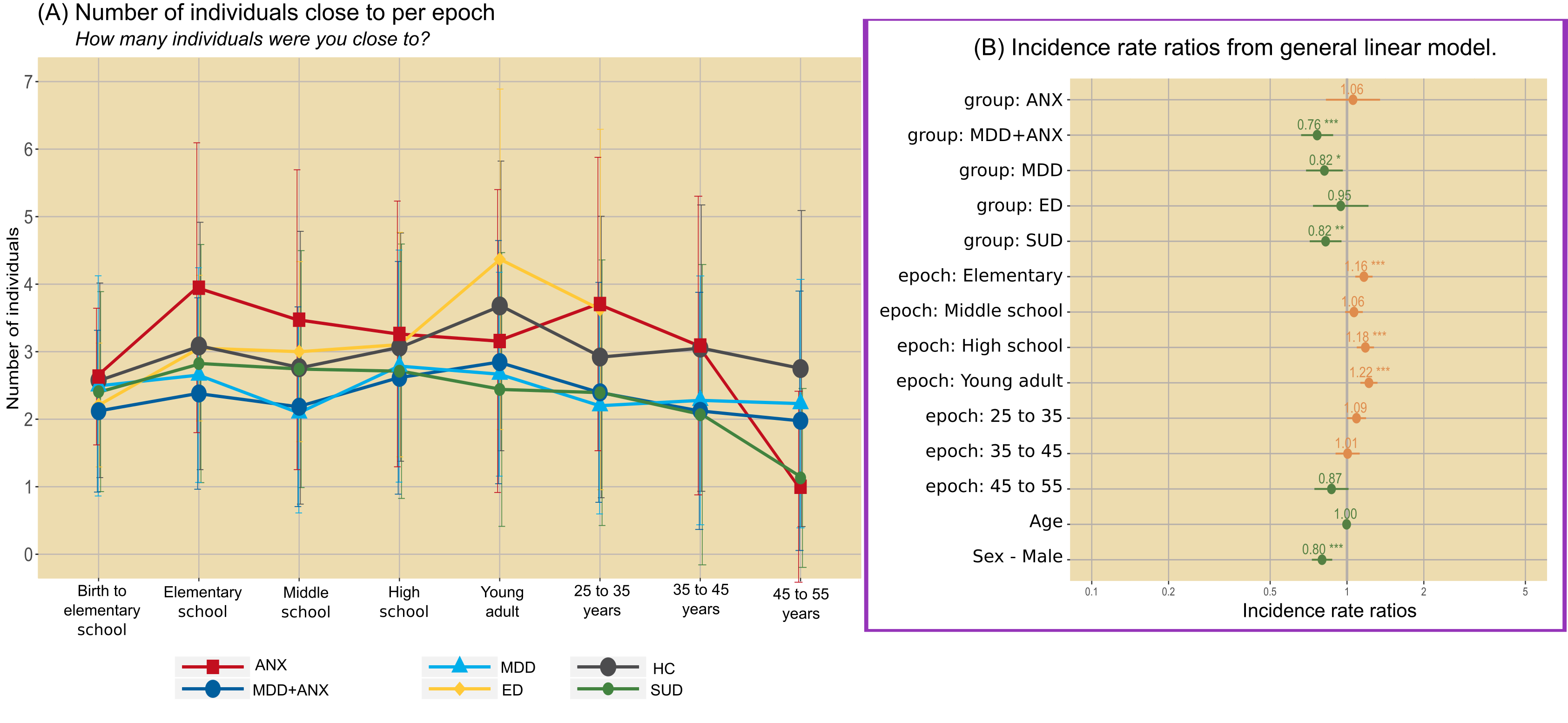


**Multimedia Appendix 3, Figure 2. Average number of people participants reported being close to, by epoch, and regression coefficients from generalized linear mixed-effects models.** On the left are the average number of people participants reported being close for each diagnostic group and epoch, with error bars representing standard deviation. On the right side are the regression coefficients obtained from the from generalized linear mixed-effects models identified by Akaike Information Criterion (AIC), with error bars representing 95% confidence intervals and p-value thresholds noted with *.05, **.01, ***.001.

**
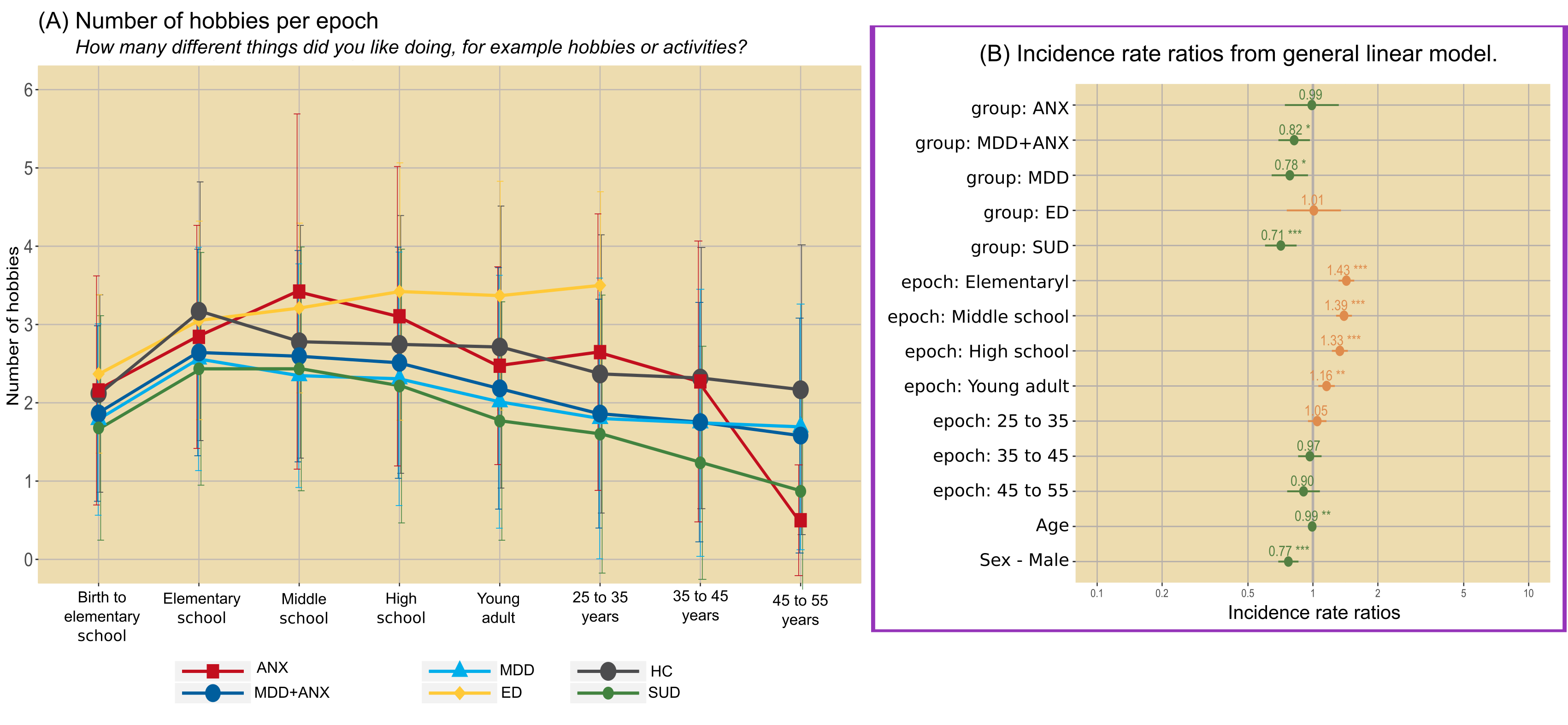
Multimedia Appendix 3, Figure 3. Average number of hobbies reported by epoch and regression coefficients from generalized linear mixed-effects models.** On the left are the average number of hobbies for each diagnostic group by epoch, with error bars representing standard deviation. On the right side are the regression coefficients obtained from the from generalized linear mixed-effects models identified by Akaike Information Criterion (AIC), with error bars representing 95% confidence intervals and p-value thresholds noted with *.05, **.01, ***.001.

**Multimedia Appendix, Table 3. Choices of GLMM model specifications for quantitative analyses**

| Outcome | Distribution | Link function | Fixed-effects | Random-effects or correlation structure† |
| --- | --- | --- | --- | --- |
| General mood rating | Gaussian | Identity | epoch  group,  age  gender  epoch-by-group interaction† | Random intercept  or  AR(1) |
| Number of good-minus-bad events | Gaussian  ZI-Gaussian |  |  |  |
| number of different types of drugs exposed to | Poisson  ZI-Poisson  negative binomial (NB) | Log |  |  |
| number of different mental health treatments received |  |  |  |  |
| number of individuals the individual reports being close to |  |  |  |  |
| number of hobbies reported |  |  |  |  |
