# Supplementary material for: Web-Based Graphic Representation of the Life Course of Mental Health: Cross-Sectional Study Across the Spectrum of Mood, Anxiety, Eating, and Substance Use Disorders
Source: JMIR Ment Health. 2020 Jan 28;7(1):e16919. doi: 10.2196/16919 (PMC7013650; doi:10.2196/16919)
Supplement: Multimedia Appendix 4 [file mental_v7i1e16919_app4.docx]

**This is a Multimedia Appendix to a full manuscript entitled “Web-based graphic representation of the life course of mental health: A cross-sectional study across the spectrum of mood, anxiety, eating, and substance use disorders.**

Additional information concerning the Tulsa Life Chart (TLC) assessment instrument and analyses used for the current study is available through open source websites. Specifically, interactive Graphs for each case, REDCap forms, and code for creating the interactive TLC graphs, are available on GitHub at <https://laureate-institute-for-brain-research.github.io/tlc/>. R code used for analysis can be found at <https://osf.io/58h9r/>.

**Supplemental Case Descriptions**

Case 2 is a 36 year old Caucasian female. She was born in 1980 and grew up in Westbrook, Maine with one younger sister. She reports that from ages 8 to 12, she was sexually abused by her uncle. At age 13, she began using alcohol (1 - 2 beers) daily and marijuana (one joint) daily and began displaying behavioral problems such as skipping school or ignoring her parents’ rules. At age 14, her parents took her to therapy due to these behavioral problems and she temporarily stopped using substances. Starting again at age 15, she continued to use alcohol (6 beers per day) and marijuana (1/4 oz per day). At age 18, her parents separated. From ages 19 to 25, she reported using alcohol (1 pint of liquor per day), marijuana (1 oz per week), heroin (2g per day), and cocaine (1g per day). She was charged with driving under the influence (DUI) for the first time at age 20 (alcohol), and then again at age 22 (alcohol) and 25 (heroin). She has been married twice, at age 25 (for two years), and 32 (for four years). Case 2 has three children; a son in 2001 (at age 20), and daughters in 2003 (at age 22) and 2006 (at age 25). Case 2’s third DUI was charged as a felony, for which she spent three months in jail. At that time, her two children were removed from her custody. Case 2 reported working as an exotic dancer from age 18 to 22 and as a bartender from age 20 to 26. At age 29, she entered inpatient treatment for substance use. She reportedly relapsed soon after completing treatment, returning to her pre-treatment level of substance use. At age 37, she again entered inpatient substance use treatment. At the time of evaluation she has not used substances for 3 months and is currently living in a transitional living facility. She is currently unemployed, and lives in Ft. Lauderdale, FL.

As can be observed within the TLC graph (Figure S2), her mood drastically decreased between high school and young adult years, and has improved slightly since age 25. She has moved between cities/states several times in her life. While she reports being close to family members at times, there is a noticeable lack of social support throughout her life and she currently cannot name anyone she is close to. She also reports no hobbies since high school and has had very few jobs, with the jobs she has had being in environments with high access/exposure to alcohol or other drugs. Her current scores on screening measures were as follows: SCOFF=0, PHQ=6, OASIS=5, and DAST=9, suggesting elevations in substance use (severe), anxiety (moderate), and depression (mild) symptoms. Through the MINI International Neuropsychiatric Inventory, she was diagnosed with recurrent MDD; and opioid, alcohol, and cannabis use disorders.

**Multimedia Appendix, Figure 4. Image from the interactive Tulsa Life Chart for Case 2, from the SUD+ diagnostic group.**

Case 3 is a 38 year old Caucasian male. He was born in 1977 and grew up in Glenpool, OK with one younger brother. Throughout childhood, his father abused alcohol and other substances, and also sold illicit drugs (including marijuana and methamphetamine). He reports being physically abused and periodically neglected by his father from ages 4 to 13. He indicated that there were times when he was locked in a closet without food for over a day. At age 19, Case 3’s father reportedly went to prison for manufacturing methamphetamine. He reports being married once at age 25 (now divorced), with two children (at age 22, and 24). He began using and selling marijuana at age 16. From high school until age 25, he was reportedly using marijuana daily (1 oz per week), alcohol once or twice per week (4-5 beers each time). He also reported often getting into fights with his classmates during high school. He married his wife at age 25 and stopped using substances. However, at age 28, he reportedly had an affair and he and his wife were divorced. He reports a precipitous decline in his mood during this time. After the divorce, he moved in with his father and started to sell marijuana and cocaine as a primary source of income. From age 28 until age 37, he indicated using opioids two or three times per week (1 pill per use), heroin intravenously daily (2g per day), and methamphetamine weekly (1 g per use). At age 30, his mother passed away from a cardiac event. At age 38, his father again went to prison. When this happened, Case 4 was homeless for over one year. In 2016 he entered into inpatient substance use treatment. He is currently living in a halfway living program.

As can be observed within the TLC graph (Figure S3), his mood did not exhibit a significant decrease until his mid 20’s, despite the fact that significant trauma and drug use occurred much earlier. His later mood decline coincided with the same epoch (age 25-35) that he married and purchased a home, as well as got divorced and began selling illicit drugs. While he reported being involved in many different organizations and hobbies during his high school years, this dropped off significantly in young adulthood, also the same epoch in which his mood declined. The people he reports being close to center around his family. Other than selling drugs, he reported only one job as a construction worker which coincided with the same time as his marriage. His scores on screening measures were as follows: SCOFF=0, PHQ=22, OASIS=11, and DAST=8, suggesting elevations in substance use (substantial), anxiety (severe), and depression (severe) symptoms. Through the MINI International Neuropsychiatric Inventory, he was diagnosed with recurrent MDD; agoraphobia; amphetamine, and opioid use disorder.

**Multimedia Appendix, Figure 5. Image from the interactive Tulsa Life Chart for Case 3, from the SUD+ diagnostic group.**

Case 4 is a 20 year-old Asian American female. She was born in 1994 and grew up in New Haven, CT as one of five children. She described herself as an anxious child, a perfectionist, and someone who always ‘sticks to the rules.’ At age 10, she began picking at her skin and pulling out her eyelashes and eyebrows to help relieve anxiety. She indicated that she also began feeling down and intermittently depressed around this age. At age 14, she began dieting, restricting her intake of food (particularly high calorie items), and exercising obsessively after one of her peers commented that she ‘looked fat’ in her swimsuit during swim practice. She reports having two major episodes of depression in 2009 (age 15) and 2012 (age 18). At age 15, she experienced several months of suicidal ideation where she had passive thoughts and plans, with no intent, about overdosing on prescription medications or wishing she would get into a fatal car accident. In 2009, she briefly went to family therapy, in part to address her eating disorder. She reported having to drop out of college at Loyola University after her first year due to severe eating disorder behaviors. She engaged in inpatient eating disorder treatment twice, once from October, 2014 to January, 2015 and again from July, 2015 to September, 2015 following a relapse of her eating disorder. Her eating disorder was in early remission at the time of evaluation. She currently lives in a transitional living facility and has recently resumed college at Northeastern State University. She has never been married and has no children.

As can be observed within the TLC graph (Figure S4), Case 4’s mood decreased, and her anxiety and eating disorder symptoms increased, between age 10 to 15, and it has improved dramatically over the past few years, coinciding with treatment engagement. Throughout her life, she reports many different hobbies and people she is close to, with minimal alcohol or drug use. Case 4’s current scores on screening measures were as follows: SCOFF=3, PHQ=1, OASIS=8, and DAST=0, suggesting elevation in eating disorder and anxiety symptomatology (moderate). Through the MINI International Neuropsychiatric Inventory, she was diagnosed with anorexia nervosa, in partial remission; and recurrent MDD.

**Multimedia Appendix, Figure 6. Image from the interactive Tulsa Life Chart for Case 4, from the ED+ diagnostic group.**
